# Chronic Spontaneous Urticaria—New Predictor on the Horizon?

**DOI:** 10.3390/jcm13226812

**Published:** 2024-11-13

**Authors:** Bartosz Bartosik, Katarzyna Kapeluszna, Dagmara Bartosik, Agata Chobot, Paulina Ciszewska-Hołda, Ewa Gawrylak-Dryja, Anna Klus, Rafał Bułdak, Zenon Brzoza

**Affiliations:** 1 Department of Internal Diseases, Allergology, Endocrinology and Gastroenterology, Institute of Medical Sciences, University of Opole, 45-040 Opole, Poland; bartosz.bartosik@uni.opole.pl (B.B.); katarzyna.kapeluszna@uni.opole.pl (K.K.); dagmara.bartosik@uni.opole.pl (D.B.); 2 Department of Pediatrics, Institute of Medical Sciences, University of Opole, 45-040 Opole, Poland; agata.chobot@uni.opole.pl (A.C.); paulina.ciszewska-holda@uni.opole.pl (P.C.-H.); 3 Department of Biochemistry and Laboratory Diagnostics, Institute of Medical Sciences, University of Opole, 45-040 Opole, Poland; ewa.gawrylak-dryja@uni.opole.pl (E.G.-D.); anna.klus@uni.opole.pl (A.K.); rafal.buldak@uni.opole.pl (R.B.)

**Keywords:** calprotectin, urticaria, angioedema, predictor, urticaria activity score, urticaria control test

## Abstract

Chronic urticaria is one of the most common diseases in allergology and dermatology practice with unclear causes of occurrence. **Background:** Some studies emphasize the correlation between inflammation in chronic urticaria and disturbed intestinal microbiota. It raises the question about the role of some intestine-related substances in the pathogenesis of urticaria as well as their potential role as disease predictors. Calprotectin is an acute-phase protein with a well-established diagnostic position in the field of gastroenterology. There are some data on the relationship between this parameter and gut microbiota. The major aim of this preliminary study is to investigate whether calprotectin can be potentially taken into account as a disease course predictor in urticaria. **Methods:** We included in our study 54 chronic spontaneous urticaria (CSU) patients (of whom 26 manifested the symptoms of recurrent angioedema) and 29 patients allergic to Hymenoptera venom for the reference group (in these patients, before venom immunotherapy induction, full diagnostics is performed including intestinal problems). Disease activity in the CSU patients was assessed using the Urticaria Activity Score (UAS) and the disease control in this group was assessed with the Urticaria Control Test (UCT). Moreover, we analyzed fecal and serum calprotectin concentrations. **Results:** Positive correlation was found only between the values of serum calprotectin concentration and the control level of CSU symptoms with the lack of other relations. **Conclusions:** Our results do not supply unequivocal evidence for calprotectin as a potential marker of CSU course, though this concept, in the light of growing evidence for microbiota’s role in urticaria, requires further research.

## 1. Introduction

Urticaria is one of the most common diseases in allergological and dermatological practice. The cause of urticaria is often unknown. Chronic spontaneous urticaria (CSU) is characterized by a spontaneous outburst of urticarial wheals recurring for more than 6 weeks, accompanied by pruritus and often the symptoms of angioedema. Urticaria is a common condition that affects people of all ages, races, and genders. However, about two-thirds of patients with CSU are women, with a peak incidence in the fourth decade of their lives, which is consistent with the epidemiology of autoimmune diseases, including thyroid diseases, type 1 diabetes, systemic lupus erythematosus, rheumatoid arthritis, vitiligo, and celiac disease [1]. Current studies emphasize the coexistence of CSU with other autoimmune diseases in about 30% of the cases [2].

The phenomenon of gut microbiome contribution to the pathogenesis of CSU is strongly supported [3]. Altered bacterial diversity noticed in CSU results in the decomposition of gut microbiota [4]. Gut microbiome changes in CSU patients were correlated with levels of inflammatory parameters, disease duration, and response to treatment [5]. The above mentioned relations result in looking for potent intestine-related substances to be taken into account when analyzing CSU pathogenesis. Calprotectin is a S100 family protein belonging to the calgranulins. It is mainly found in the cytoplasm of neutrophils and monocytes. Upon the activation of neutrophils or monocytes by pro-inflammatory cytokines, calprotectin is released into the extracellular space. Participation in the inflammatory process is also associated with chemotactic properties towards neutrophils. It also demonstrates the ability to induce apoptosis and the synthesis of immunoglobulins by plasma cells and other mediators involved in the inflammatory reaction [6]. One of the recently described phenomena involving calprotectin is NETosis—a non-specific immune response that is based on the disintegration of the neutrophil cell membrane and the decondensation of DNA in response to an infection [7,8]. These properties indicate an important role of this protein in the cascade of inflammatory processes. It is a substance that occurs physiologically in serum, saliva, amniotic fluid, and other body fluids and secretions [9]. The normal serum levels of calprotectin are estimated to range from 0.1 to 1.6 μg/mL, and can be elevated in numerous conditions such as infection, inflammation, or cancer. Determining the concentration of this protein can facilitate differentiating physiological and pathological conditions [10]. The first studies on this subject come from the beginning of the 1990s [11]. The following years brought further evidence on the diagnostic usefulness of assessing the presence of this protein in the stool in patients with inflammatory bowel diseases (IBDs) [12,13]. An increased concentration of calprotectin in the stool has a prognostic value and is a very sensitive sign of exacerbation in patients with Crohn’s disease (CD) or ulcerative colitis (UC) in clinical remission [14]. Assessing the presence of calprotectin in the stool may be useful in detecting an endoscopic recurrence of CD in asymptomatic patients after surgery [15]. Determining the calprotectin concentration also comes with some limitations [16,17]. Currently, it is recognized that copromarkers should be used mainly in the early diagnosis of patients with symptoms coming from the gastrointestinal tract, for whom a decision should be made to perform endoscopic examinations [18].

Calprotectin is supposed to be a potential biomarker for monitoring disease activity in rheumatoid arthritis patients or axial spondyloarthritis. The role of calprotectin is valuable when C-reactive protein (CRP) is normal or difficult to interpret, such as in patients treated with medications that suppress IL-6. No associations between serum calprotectin levels and disease activity in psoriatic arthritis were noticed [19]. Additionally, calprotectin is analyzed as a potential new biomarker for disease severity in idiopathic pulmonary fibrosis (IPF). Its serum levels are significantly increased in patients with IPF compared with healthy controls and correlate with clinical and spirometry parameters [20]. Serum calprotectin levels are also increased in acne vulgaris patients compared to healthy control subjects and are positively correlated with disease severity [21].

In the literature, the correlation between the occurrence and course of urticaria and numerous disorders of the gastrointestinal tract is mentioned. An association between CSU and gastroesophageal reflux is suggested [22]. Similarly, gastritis and Helicobacter pylori infection have been proven to play a role in CSU pathogenesis [23,24]. At the same time, the search for a marker that would allow predicting the occurrence of urticaria and monitoring the disease activity is ongoing. So far, no parameter has been found that fulfills this role. Could calprotectin be one of such predictors? As a protein directly involved in the cascade of inflammatory processes, present not only in the stool but also in the serum, it gives great hope to be included in the observational process in patients with chronic urticaria. In this study, we try to answer the question of whether calprotectin can be considered as a marker correlating with disease activity and severity.

## 2. Materials and Methods

### 2.1. Study Group

Consecutive patients with CSU and patients allergic to Hymenoptera venom for the reference group treated at the Department of Internal Diseases, Allergology, Endocrinology and Gastroenterology of the University Hospital in Opole in 2019–2021 were invited to join this study. We used Hymenoptera allergic patients in the reference group as in these patients, before venom immunotherapy (VIT) induction, full general diagnostics is performed to diagnose other disorders and immunological abnormalities including intestinal problems.

### 2.2. Laboratory Methods

Calprotectin concentration in the serum and feces was determined in all the patients (Human calprotectin ELISA kit, EKX-D79893, Nordic Biosite, Täby, Sweden). This kit is based on sandwich enzyme-linked immune-sorbent assay technology. Anti-calprotectin antibody was pre-coated onto 96-well plates and the biotin-conjugated anti-calprotectin antibody was used as a detection antibody. Test sensitivity was <9.375 ng/mL and range was 15.63–1000 ng/mL.

### 2.3. Disease Activity and Control Assessment

Disease activity in the CSU group was assessed using the Urticaria Activity Score (UAS)—a tool that allows the daily assessment of the itching intensity and the number of urticarial wheals on a scale of 0–6; the score summed up over 7 consecutive days is assessed (UAS7, a maximum of 42 points can be achieved—a greater number of points indicates the greater activity of the disease). The level of disease control in this group was assessed with the Urticaria Control Test (UCT)—this tool consists of 4 questions on the basis of which the final score is calculated within the range of 0–16 points, where higher values indicate a better control of the disease symptoms [25]. Moreover, the coexistence of angioedema symptoms was assessed in the CSU group.

### 2.4. Study Group Characteristics

The patients with CSU were treated with antihistamines and four of them additionally with omalizumab. The group of patients with an allergic reaction to insect bites included the patients with overcome Miller’s grade III or IV anaphylactic reaction (intensity of allergic reaction requiring further diagnostics and potent therapy)—they were subjected to allergen immunotherapy.

### 2.5. Statistical Analysis

The Mann–Whitney U test and Spearman’s Rank Correlation test (Statistica 13.3, Statsoft Inc., Tulsa, OK, USA) were used for the statistical analysis. The results were considered significant at a *p*-value lower than 0.05.

## 3. Results

**Figure 1:**
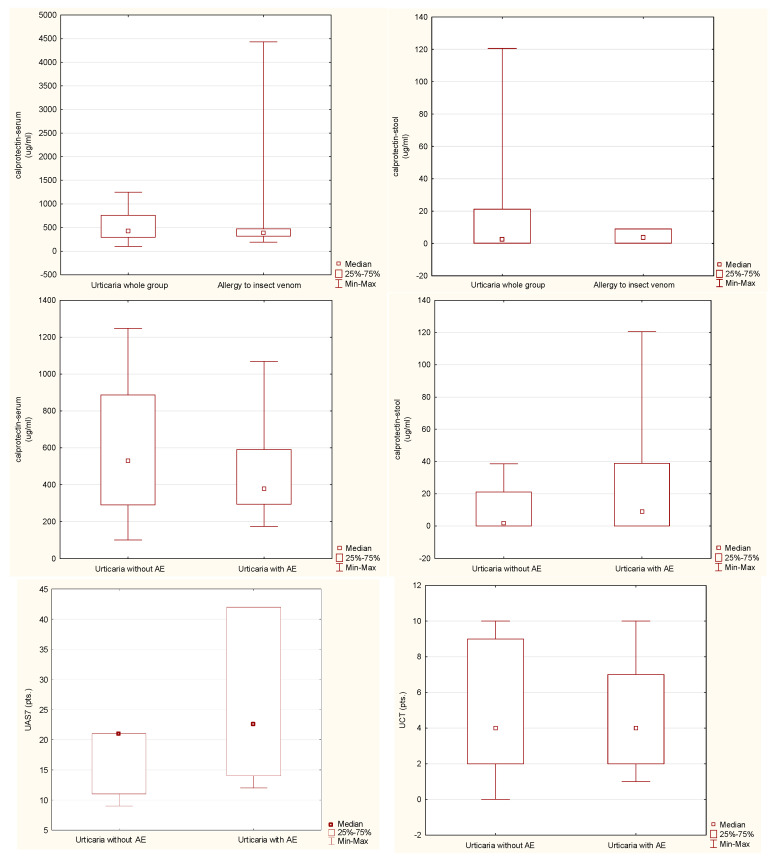
Analyzed parameter levels in studied groups (AE, angioedema; UAS7, Urticaria Activity Score 7-day assessment; UCT, Urticaria Control Test).

The CSU group included 54 patients, of whom 26 (48%) manifested the symptoms of recurrent angioedema. The reference group included 29 patients allergic to Hymenoptera venom (Table 1). No differences in the concentration of calprotectin in both feces and serum were found when comparing the group of patients with CSU (as a whole) with the insect venom allergy group (Table 2, Figure 1). Such differences were also not demonstrated in the comparison of the subgroups with and without angioedema within the CSU group (Table 3, Figure 1). There were no differences between the groups with and without angioedema in the disease activity and control based on UAS7 and UCT, respectively (Table 3, Figure 1). A statistically significant positive correlation was found between the values of serum calprotectin concentration and the control level of the disease symptoms, assessed with the UCT scale in the urticaria whole group and urticaria without angioedema. There were no similar links for the concentration of calprotectin in the feces, nor links for the correlation of these parameters with the UAS7 disease activity assessment scale (Table 4).

## 4. Discussion

Calprotectin, an acute-phase protein, is a part of the cytoplasmic granules of neutrophils and, to a lesser extent, of the monocytic lineage cells. The concentration of this copromarker is associated with inflammation activity in the gastrointestinal tract; however, there is a number of other diseases that may cause an increase in its concentration. Some new studies showed increased levels of serum calprotectin in rheumatological, pulmonological, or dermatological diseases. In addition, an increased level of serum calprotectin is linked with disease activity or severity [19,20,21]. The biological function of calprotectin is not fully understood. An important feature of calprotectin is bacteriostatic and fungicidal [16]. It is also believed that it may play the role of a chemotactic factor in an inflammatory response and stimulate the synthesis of immunoglobulins. In the gastrointestinal tract, it is released under the influence of neutrophil activation, and its concentration in the feces is proportional to the number of neutrophils present in the mucosa. Determining the concentration of this protein facilitates differentiating the physiological and pathological conditions [10]. Currently, calprotectin has an established position in the diagnosis and monitoring of inflammatory bowel diseases. An important feature of the discussed protein from the point of view of laboratory diagnostics is its high biological stability—according to some reports, it does not decompose for up to 7 days at room temperature [6]. According to a wide spectrum of data, gut microbiota imbalance is supposed to contribute to the pathogenesis of numerous chronic diseases including inflammatory bowel diseases. On the other hand, the role of microbiota in CSU is still unclear, though its inflammatory pathogenesis is undoubted [26,27].

From the point of view of clinical practice, the problem in chronic urticaria is the lack of reliable parameters that would be useful in predicting the course and response to the treatment of this disease. In recent years, various studies have attempted to identify substances that would fulfill the requirements as a biomarker of the disease, which would significantly facilitate the course monitoring and treatment of urticaria. No such reliable and useful parameter completing all the necessary criteria has been found to date [28]. The study by Song Y et al. emphasizes the correlation between inflammation in chronic urticaria and disturbed intestinal microbiota (including inflammation in the gastrointestinal tract measured by the level of calprotectin in the stool) [5]. We can speculate that the gut microbiome alterations noticed in CSU can potentially contribute to inflammation related to CSU pathogenesis [29]. It has even been suggested that in the future, therapies aimed at changing the intestinal microbiota could potentially be a chance for the effective treatment of chronic urticaria. According to the current literature, the correlation of calprotectin concentration as a marker (in both feces and serum) in the diagnosis and treatment of chronic urticaria has not been directly assessed and we are the first to analyze this concept directly. The only statistically significant finding of our study is a positive correlation between the values of serum calprotectin concentration and the control level of the disease symptoms as assessed with UCT. This seemingly paradoxical phenomenon may be explained by the possible suppression of mast cells by calgranulins [30]. This mechanism could explain why a slightly higher, yet normal, level of calprotectin correlates with the control of the disease symptoms. Another issue is no influence of angioedema on calprotectin levels. In general, this fact is not surprising concerning the similar pathophysiology of wheal and angioedema occurrence in the course of CSU. On the other hand, we did not notice any significant correlation between urticaria activity as assessed with UAS7 and calprotectin levels. This finding certifies against the possible function of calprotectin as a CSU biomarker, though we must be aware that dependence between UAS7 and UCT is high but not complete [25]. Altogether, our results do not bring unequivocal evidence for calprotectin as a novel CSU biomarker.

So far, the studies have mainly assessed the concentration of calprotectin in the stool. Our study shows that serum calprotectin should also be considered. Generally, it has been shown that the major extracellular effect of calprotectin is to amplify the inflammatory process through several mechanisms. The concentration of this protein in other body fluids, e.g., saliva and bronchial secretions, should also be considered. Of course, this requires further research, a larger population of patients, and the development of standards and high-quality tests. Additionally, differences between chronic urticaria patients with and without coexisting angioedema should be further explored.

The limitation of our study is that we did not analyze the evolution of the calprotectin levels and its relation to parameters characterizing disorder course over time.

## 5. Conclusions

The well-established diagnostic position of calprotectin potentially gives hope for the use of this marker to monitor symptoms in different disorders. The result of our study points to a potential role in CSU, though our findings are unequivocal. This issue requires further research and the development of uniform standards of conduct.

## Figures and Tables

**Table 1:** Demographic data of studied groups (AE, angioedema).

	Allergy to Insect Venom	Urticaria Whole Group	Urticaria with AE	Urticaria Without AE
Female	18	39	18	21
Male	11	15	8	7
Mean age	49.7	46.6	52.4	40.7
Age range	19–71	18–80	30–80	18–63

**Table 2:** Serum and fecal calprotectin concentration values in the group of patients with chronic spontaneous urticaria and in the group of patients allergic to insect venom (min, minimum; max, maximum; *p*, probability level).

	Allergy to Insect Venom			Urticaria Whole Group			*p*
	Max	Min	Median	Max	Min	Median	
Calprotectin— serum (µg/mL)	4434	189	387	1248	101	426	>0.05
Calprotectin— stool (µg/mL)	8.96	0.10	3.67	120.6	0.1	2.55	>0.05

**Table 3:** Serum and fecal calprotectin concentrations in the group of patients with chronic spontaneous urticaria with and without angioedema (AE, angioedema; UAS7, Urticaria Activity Score 7-day assessment; UCT, Urticaria Control Test; min, minimum; max, maximum; *p*, probability level).

	Urticaria Without AE			Urticaria with AE			*p*
	Max	Min	Median	Max	Min	Median	
Calprotectin—serum (µg/mL)	1248	101	531	1068	174	379.5	>0.05
Calprotectin—stool (µg/mL)	38.68	0.10	1.76	120	0.10	9.07	>0.05
UAS7 (pts.)	21.0	9.00	21.0	42.0	12.0	22.5	>0.05
UCT (pts.)	10.0	0.00	4.00	10.0	1.00	4.00	>0.05

**Table 4:** Correlations of the serum and fecal calprotectin concentration with the parameters of the disease’s activity and control levels in the group of patients with chronic spontaneous urticaria and in the subgroups depending on the coexistence of angioedema (AE, angioedema; UAS7, Urticaria Activity Score 7-day assessment; UCT, Urticaria Control Test; R, Spearman’s correlation coefficient; *p*, probability level; pts., points).

	Urticaria Without AE		Urticaria with AE		Urticaria Whole Group	
	*p*	R	*p*	R	*p*	R
calprotectin— serum and UAS7	0.72	−0.22	0.83	0.07	0.67	0.11
calprotectin— serum and UCT	0.01	0.86	0.23	0.34	0.03	0.47
calprotectin—stool and UAS7	0.57	−0.34	0.55	0.31	0.92	0.04
calprotectin—stool and UCT	0.52	0.30	0.64	−0.22	0.72	0.11

## Data Availability

Dataset available on request from the authors.

## References

[1] Kolkhir P., Borzova E., Grattan C., Asero R., Pogorelov D., Maurer M. (2016). Autoimmune comorbidity in chronic spontaneous urticaria: A systematic review. Clin. Exp. Allergy.

[2] Gonzalez-Diaz S.N., Sanchez-Borges M., Rangel-Gonzalez D.M., Guzman-Avilan R.I., Canseco-Villarreal J.I., Arias-Cruz A. (2020). Chronic urticaria and thyroid pathology. World Allergy Organ. J..

[3] Krišto M., Lugović-Mihić L., Muñoz M., Rupnik M., Mahnic A., Ozretić P., Jaganjac M., Ćesić D., Kuna M. (2023). Gut microbiome composition in patients with chronic urticaria: A review of current evidence and data. Life.

[4] Wang D., Guo S., He H., Gong L., Cui H. (2020). Gut microbiome and serum metabolome analyses identify unsaturated fatty acids and butanoate metabolism induced by gut microbiota in patients with chronic spontaneous urticaria. Front. Cell Infect. Microbiol..

[5] Song Y., Dan K., Yao Z., Yang X., Chen B., Hao F. (2022). Altered gut microbiota in H1-antihistamine-resistant chronic spontaneous urticaria associates with systemic inflammation. Front. Cell Infect. Microbiol..

[6] Walsham N.E., Sherwood R.A. (2016). Fecal calprotectin ininflammatory bowel disease. Clin. Exp. Gastroenterol..

[7] Delgado-Rizo V., Martínez-Guzmán M.A., Iñiguez Gutierrez L., García-Orozco A., Alvarado-Navarro A., Fafutis-Morris M. (2017). Neutrophil extracellular traps and its implications in inflammation: An overview. Front. Immunol..

[8] Eder P. (2018). Przydatność biomarkerów w ocenie aktywności nieswoistych chorób zapalnych jelit—Wskazówki praktyczne. Gastroenterol. Klin..

[9] Kosiara M., Paradowski L. (2008). Kalprotektyna. Gastroenterol. Pol..

[10] Johne B., Fagerhol M.K., Lyberg T., Prydz H., Brandtzaeg P., Naess-Andresen C.F., Dale I. (1997). Functional and clinical aspects of the myelomonocyte protein calprotectin. Mol. Pathol..

[11] Roseth A.G., Fagerhol M.K., Aadland E., Schjønsby H. (1992). Assesment of the neutrophil dominanting protein calprotectin in feces. A methodologic study. Scand. J. Gastroenterol..

[12] Sipponen T., Savilahti E., Kolho K.L., Nuutinen H., Turunen U., Färkkilä M. (2008). Crohn’s disease activity assessed by fecal calprotectin and lactoferrin: Correlation with Crohn’s disease activity index and endoscopic findings. Inflamm. Bowel Dis..

[13] Sipponen T., Karkkainen P., Savilahti E., Kolho K.L., Nuutinen H., Turunen U., Färkkilä M. (2008). Correlation of faecal calprotectin and lactoferrin with an endoscopic score for Cronh’s disease and histological findings. Aliment. Pharmacol. Ther..

[14] Gisbert J.P., Bermejo F., Perez-Calle J.L., Taxonera C., Vera I., McNicholl A.G., Algaba A., López P., López-Palacios N., Calvo M. (2009). Fecal calprotectin and lactoferrin for the prediction of inflammatory bowel disease relapse. Inflamm. Bowel Dis..

[15] Orlando A., Modesto I., Castiglione F., Scala L., Scimeca D., Rispo A., Teresi S., Mocciaro F., Criscuoli V., Marrone C. (2006). The role of calprotectin in predicting endoscopic post-surgical recurrence in asymptomatic Crohn’s disease: A comparison with ultrasound. Eur. Rev. Med. Pharmacol. Sci..

[16] von Roon A.C., Karamountzos L., Purkayastha S. (2007). Diagnostic precision of fecal calprotectin for inflammatory bowel disease and colorectal malignancy. Am. J. Gastroenterol..

[17] Tibble J.A., Sigthorsson G., Foster R., Scott D., Fagerhol M.K., Roseth A., Bjarnason I. (1999). High prevalence of NSAID enteropathy as shown by a simple faecal test. Gut.

[18] Van Rheenen P.F., Van de Vijver E., Fidler V. (2010). Faecal calprotectin for screening of patients with suspected inflammatory bowel disease: Diagnostic meta-analysis. BMJ.

[19] Jarlborg M., Courvoisier D.S., Lamacchia C., Martinez Prat L., Mahler M., Bentow C., Finckh A., Gabay C., Nissen M.J., Physicians of the Swiss Clinical Quality Management (SCQM) Registry (2020). Serum calprotectin: A promising biomarker in rheumatoid arthritis and axial spondyloarthritis. Arthritis Res. Ther..

[20] Machahua C., Guler S.A., Horn M.P., Planas-Cerezales L., Montes-Worboys A., Geiser T.K., Molina-Molina M., Funke-Chambour M. (2021). Serum calprotectin as new biomarker for disease severity in idiopathic pulmonary fibrosis: A cross-sectional study in two independent cohorts. BMJ Open Respir. Res..

[21] Korkmaz S., Fıçıcıoğlu S.K. (2018). Calprotectin can play an inflammatory role in acne vulgaris. Adv. Dermatol. Allergol..

[22] Abadeh A., Herman S.M., Abdalian R. (2023). The prevalence of gastrointestinal symptoms and cobalamin deficiency in patients with chronic urticaria. Allergy Asthma Clin. Immunol..

[23] Zheleznov S., Urzhumtseva G., Petrova N., Sarsaniia Z., Didkovskii N., Dörr T., Zuberbier T. (2018). Gastritis Can Cause and Trigger Chronic Spontaneous Urticaria Independent of the Presence of *Helicobacter pylori*. Int. Arch. Allergy Immunol..

[24] Chu C.Y., Zuberbier T. (2020). Urticaria and the gut. Curr. Opin. Allergy Clin. Immunol..

[25] Brzoza Z., Badura-Brzoza K., Maurer M., Hawro T., Weller K. (2022). Chronic spontaneous urticaria activity, impact and control as well as their changes are strongly linked, and these links are not affected by angioedema or comorbid inducible urticaria—Results from the validation of the Polish Urticaria Control Test. World Allergy Organ. J..

[26] Zhu L., Jian X., Zhou B., Liu R., Muñoz M., Sun W., Xie L., Chen X., Peng C., Maurer M. (2024). Gut microbiota facilitate chronic spontaneous urticaria. Nat. Commun..

[27] Gomułka K., Mędrala W. (2022). Serum Levels of Vascular Endothelial Growth Factor, Platelet Activating Factor and Eosinophil-Derived Neurotoxin in Chronic Spontaneous Urticaria—A Pilot Study in Adult Patients. Int. J. Mol. Sci..

[28] Zuberbier T., Abdul Latiff A.H., Abuzakouk M., Aquilina S., Asero R., Baker D., Ballmer-Weber B., Bangert C., Ben-Shoshan M., Bernstein J.A. (2022). The international EAACI/GA^2^LEN/EuroGuiDerm/APAAACI guideline for the definition, classification, diagnosis, and management of urticaria. Allergy.

[29] Al Bander Z., Nitert M.D., Mousa A., Naderpoor N. (2020). The Gut Microbiota and Inflammation: An Overview. Int. J. Environ. Res. Public Health.

[30] Goyette J., Geczy C.L. (2011). Inflammation-associated S100 proteins: New mechanisms that regulate function. Amino Acids.

